# Нейроэндокринная регуляция репродуктивной системы: интеграция исследований нейропептидов и экспериментальной патофизиологии аменореи различного генеза (обзор литературы)

**DOI:** 10.14341/probl13641

**Published:** 2026-01-28

**Authors:** Ю. С. Евсеева, Ю. С. Абсатарова, Е. Н. Андреева, В. А. Иоутси, С. А. Румянцев, Е. В. Шереметьева, О. Р. Григорян, Г. А. Мельниченко

**Affiliations:** Национальный медицинский исследовательский центр эндокринологии им. академика И.И. ДедоваРоссия; Endocrinology Research CentreRussian Federation; Национальный медицинский исследовательский центр эндокринологии им. академика И.И. Дедова; Российский университет медициныРоссия; Endocrinology Research Centre; Russian University of MedicineRussian Federation; Национальный медицинский исследовательский центр эндокринологии им. академика И.И. Дедова; Российский национальный исследовательский медицинский университет имени Н.И. ПироговаРоссия; Endocrinology Research Centre; Pirogov National Research Medical UniversityRussian Federation

**Keywords:** Аменорея, преждевременная недостаточность яичников, функциональная гипоталамическая аменорея, синдром поликистозных яичников, бесплодие, кисспептин, нейрокинин В, орексин А, нейротрофический фактор роста, эксперимент, Amenorrhea, premature ovarian insufficiency, functional hypothalamic amenorrhea, polycystic ovary syndrome, infertility, kisspeptin, neurokinin В, orexin А, neurotrophic growth factor, experiment

## Abstract

Нейроэндокринная регуляция репродуктивной функции представляет собой сложную систему, основанную на интеграции сигналов между центральной нервной системой и периферическими органами. Особое внимание в последние годы уделяется роли нейропептидов (кисспептин, нейротрофический фактор мозга (BDNF) и орексины), в патогенезе заболеваний, сопровождающихся нарушениями менструального цикла. В обзоре подробно рассматриваются молекулярные механизмы нейропептидной регуляции при функциональной гипоталамической аменорее (ФГА), преждевременной недостаточности яичников (ПНЯ) и синдроме поликистозных яичников (СПЯ).

Представлены данные исследований последних лет с использованием экспериментальных моделей, включающие стресс-индуцированные формы стойкого отсутствия эстрального цикла у лабораторных животных, а также имитации патологий СПЯ и ПНЯ, основанные на диетических и фармакологических воздействиях, соответственно. Дополнительно подробно рассмотрены и изучены публикации, демонстрирующие значимую роль нарушений нейропептидной передачи в патогенезе репродуктивных расстройств у женщин.

Интеграция фундаментальных исследований с клинической практикой не только способствует более глубокому пониманию патофизиологии аменореи, но и открывает широкие перспективы для разработки новых терапевтических подходов, таких как использование агонистов кисспептина или других препаратов, направленных на восстановление репродуктивной функции у женщин с различными формами менструальных дисфункций.

## Abstract (en)

Neuroendocrine regulation of reproductive function represents a complex system based on the integration of signals between the central nervous system and peripheral organs. In recent years, particular attention has been given to the role of neuropeptides — such as kisspeptin, brain-derived neurotrophic factor (BDNF), and orexins — in the pathogenesis of disorders associated with menstrual irregularities. This review provides a detailed analysis of the molecular mechanisms underlying neuropeptide regulation in functional hypothalamic amenorrhea (FHA), primary ovarian insufficiency (POI), and polycystic ovary syndrome (PCOS).

Recent experimental studies are summarized, including stress-induced models of persistent estrous cycle arrest in laboratory animals and simulation of PCOS and POI using dietary and pharmacological interventions, respectively. Additionally, the review highlights publications demonstrating the significant role of impaired neuropeptide signaling in the development of reproductive disorders in women.

The integration of fundamental research with clinical practice not only enhances our understanding of the pathophysiology of amenorrhea but also opens promising avenues for the development of novel therapeutic strategies, such as the use of kisspeptin agonists or other agents aimed at restoring reproductive function in women with various forms of menstrual dysfunction.

## ВВЕДЕНИЕ

Регулярный менструальный цикл представляет собой высокочувствительный индикатор состояния нейроэндокринной системы и служит ключевым маркером репродуктивного здоровья женщин. В настоящее время наблюдается рост частоты менструальных дисфункций, что оказывает неблагоприятное влияние на фертильность и обуславливает растущую научную и клиническую актуальность данной проблемы.

В период 2018–2022 гг. средний возраст женщин в России при рождении первого ребенка составил 27 лет, второго — 31 год. По сравнению с демографическими показателями тридцатилетней давности (20 и 21 год, соответственно), прослеживается отчетливая тенденция к отсрочке материнства. Кроме того, современные социально-экономические изменения, в частности, смещение сроков вступления в брак, рост значимости профессиональной самореализации, а также увеличение распространенности нарушений менструального цикла, ожидаемо будут способствовать дальнейшему более позднему материнству [1].

Аменорея, представляя собой не самостоятельное заболевание, а клинический симптом множества патологий, привлекает все большее внимание исследователей и клиницистов, поскольку является одной из наиболее распространенных и значимых причин женского бесплодия. Это расстройство имеет важное клиническое значение, поскольку непосредственно влияет на репродуктивное здоровье женщины, снижает шансы на естественное зачатие и требует своевременной диагностики и специализированной терапии. Учитывая высокую распространенность и многообразие форм менструальных дисфункций, углубленное изучение механизмов их развития и поиск эффективных методов терапии остаются приоритетными задачами современной медицины. Наиболее распространенными причинами аменореи на сегодняшний день считаются функциональная гипоталамическая аменорея (ФГА), синдром поликистозных яичников (СПЯ), а также преждевременная недостаточность яичников (ПНЯ) [2].

Современные исследования все убедительнее подтверждают ключевую роль нейропептидов в нейроэндокринной регуляции репродуктивной функции [3][4]. С клинической точки зрения особое значение приобретают работы, направленные на выяснение патогенетических механизмов нарушений нейропептидной передачи при длительном отсутствии менструаций различного генеза, что открывает перспективы для более точной диагностики и таргетной терапии.

ФГА представляет собой обратимое состояние, связанное с нарушением регуляции на уровне гипоталамо-гипофизарной оси и характеризующееся снижением продукции гонадотропинов и эстрогенов. Согласно статистическим данным эта патология выявляется примерно в 25–35% случаев вторичной аменореи [5]. СПЯ — наиболее частое многофакторное эндокринное нарушение у женщин репродуктивного возраста, характеризующееся гиперандрогенией, нарушением овуляции и встречающееся у 17,4–52% пациенток с нарушениями менструального цикла [6]. ПНЯ, сопровождающаяся выраженным снижением овариального резерва, также является частой причиной менструальной дисфункции; распространенность в популяции достигает 3,5% [7]. Вышеописанные нозологии, охватывающие значительную часть женского населения фертильного возраста, не только ухудшают репродуктивную функцию, но и способствуют развитию сопутствующей соматической и психоэмоциональной комордидности. Несвоевременная диагностика и лечение данных патологий могут усугубить их течение и привести к стойкому бесплодию.

Одним из ключевых направлений современной нейроэндокринологии является изучение нейропептидных систем в регуляции секреции гонадотропин-рилизинг гормона (ГнРГ). Пристальное внимание уделяется так называемым KNDy-нейронам, секретирующим кисспептин, нейрокинин В и динорфин. Эти нейроны формируют высокоспециализированную регуляторную сеть, обеспечивающую пульсирующий выброс ГнРГ. Установлено, что нейрокинин В стимулирует, а динорфин, напротив, подавляет высвобождение кисспептина, осуществляя, таким образом, тонкую аутосинаптическую регуляцию гонадотропной функции и активируя соответствующие рецепторы — NK3R (рецептор нейрокинина 3) и KOR (κ-опиоидный рецептор) [8].

Несмотря на значительный прогресс в изучении отдельных аспектов этих процессов, существующие сведения остаются фрагментарными и требуют дальнейшей систематизации, что и обусловливает высокую актуальность более углубленного изучения нейропептидов, как потенциальных диагностических маркеров и терапевтических мишеней у пациенток с нарушениями менструального цикла.

Создание экспериментальных моделей нарушений эстрального цикла на лабораторных животных является важным этапом в изучении механизмов развития и путей коррекции репродуктивных расстройств. Для моделирования аменореи используют различные подходы, включая стресс-индуцированное воздействие, диетическое ограничение, применение фармакологических препаратов и хирургическое вмешательство, позволяющие воспроизводить отклонения, возникающие на разных уровнях гипоталамо-гипофизарно-яичниковой оси.

Данные модели не только открывают возможности для детального изучения нейропептидов в гормональной регуляции репродуктивной системы, но и способствуют разработке эффективных диагностических и терапевтических подходов, что приобретает особую актуальность в контексте социально значимых заболеваний — СПЯ, ФГА и ПНЯ.

## СОВРЕМЕННЫЕ ПРЕДСТАВЛЕНИЯ О РОЛИ НЕЙРОПЕПТИДОВ В ПАТОГЕНЕЗЕ ЗАБОЛЕВАНИЙ РЕПРОДУКТИВНОЙ СИСТЕМЫ

## Кисспептин — ключевой нейропептид репродуктивной системы

Кисспептин представляет собой ключевой регулятор нейроэндокринного контроля репродуктивной функции, выступая своеобразным «дирижером» всей гипоталамо-гипофизарно-гонадной оси. Одним из центральных элементов данной системы являются ГнРГ-нейроны, локализованные в гипоталамусе и обеспечивающие ритмичное выделение гонадотропинов гипофизом, тем ­самым стимулируя работу половых желез. Их активность напрямую зависит от действия кисспептина — нейропептида, синтезируемого специализированными нейронами, сосредоточенными преимущественно в преоптической области и инфундибулярном ядре гипоталамуса. В последнем расположены KNDy-нейроны, экспрессирующие кроме кисспептина еще и нейрокинин В, и динорфин. Их скоординированная активность определяет характер импульсной секреции ГнРГ и лютеинизирующего гормона (ЛГ). Нейроны преоптической области, играют критическую роль в реализации положительной обратной связи, когда растущий фолликул выбрасывает максимальную концентрацию эстрадиола, приводя к преовуляторному пику ЛГ [8]. Помимо центрального действия, кисспептин и его рецептор (KISS1R) также экспрессируются в фолликулах, однако их локальное значение остается недостаточно изученным. Несмотря на активное накопление знаний о гипоталамической кисспептиновой сигнальной системе за последние три десятилетия, функции этого нейропептида в периферических звеньях репродуктивной регуляции, особенно в овариальной и эндометриальной ткани, по-прежнему требуют дальнейшего глубокого изучения.

В литературе описаны противоречивые результаты относительно клеточной локализации этих молекул. Представляют значительный интерес исследования, показывающие, что экспрессия гена Kiss1 преимущественно наблюдается в гранулезных клетках фолликулов, в то время как KISS1R локализуется преимущественно в ооцитах [9][10]. Эти данные свидетельствуют о наличии паракринного механизма взаимодействия между фолликулярными клетками и ооцитами, опосредованного кисспептиновой системой, активность которой регулируют эстрогеновые рецепторы типа β. Возможно, внутриовариальный нейропептид выполняет роль локального модулятора, способствуя созреванию ооцитов и овуляции и напрямую воздействуя на рецепторный аппарат яйцеклетки [11]. Локальная кисспептиновая сигнальная система, функционирующая вне зависимости от гипоталамического влияния, открывает новые перспективы для понимания внутриклеточных регуляторных путей, лежащих в основе фолликулогенеза, и подчеркивает потенциал данной молекулы в качестве биомаркера зрелости ооцита и терапевтической мишени при овуляторных нарушениях.

Проведены исследования о роли кисспептина при патологиях женской половой системы. Хотя количественные характеристики нейропептида можно использовать для диагностики, следует отметить, что их сложно точно обнаружить при низких концентрациях с использованием современных методов измерения из-за короткого периода полураспада, что ограничивает их потенциальное клиническое применение [12-14].

ФГА — одна из наиболее распространенных причин вторичной аменореи, приводящая к стойкому отсутствию самостоятельных менструаций и бесплодию. Это состояние гипоэстрогении, которое повышает риск сердечно-сосудистых заболеваний и снижает минеральную плотность костной ткани. ФГА характеризуется приобретенным подавлением физиологической пульсирующей секреции ГнРГ гипоталамусом при отсутствии идентифицируемой структурной причины и возникает в результате энергодефицита из-за чрезмерных физических нагрузок, расстройств пищевого поведения, часто в сочетании с психологическим стрессом на фоне генетической предрасположенности, что приводит сначала к функциональному нормогонадотропному, а далее при сохранении негативных факторов — гипогонадотропному гипогонадизму [15].

Сообщается, что у женщин с ФГА наблюдаются более низкие уровни кисспептина в сыворотке крови (0,17±0,11 нг/мл), чем у здоровых женщин контрольной группы (0,3±0,36 нг/мл) [16]. У пациенток с ФГА выявлена отрицательная корреляция между уровнем данного нейропептида и концентрацией кортизола в сыворотке крови. Дополнительно установлено, что при гипоталамической аменорее показатели циркулирующего кисспептина снижены, при этом особенно низкие значения наблюдаются у пациенток с уровнем ЛГ 1,3±0,1 мМЕ/мл по сравнению с пациентками с нормальными уровнями ЛГ (3,3±0,4 мМЕ/мл) [17]. У женщин с ФГА, помимо снижения уровня изучаемого нейропептида, выявлено уменьшение концентрации нейрокинина В и повышение кортикотропин-рилизинг-гормона, что может свидетельствовать о нарушениях, затрагивающих нейропептидную регуляцию и гипоталамо-гипофизарно-надпочечниковую ось [18]. При этом экспрессия рецепторов кортикотропин-рилизинг-гормона и глюкокортикоидов в KNDy-нейронах инфундибулярного ядра подтверждает их возможную роль как посредников между стресс-индуцированными сигналами и регуляцией гонадной функции [19]. Это доказано в исследовании, в котором у самок мышей после овариэктомии наблюдалось снижение активации KNDy-нейронов аркуатного ядра (аналог инфундибулярного ядра у людей) после воздействия психосоциального стресса, индуцируемого в соответствии со стандартизированными протоколами. Аналогичным образом, воздействие различных неблагоприятных факторов снижало экспрессию Kiss1 в гипоталамусе у самок мышей. Кроме того, введение кортикотропин-рилизинг гормона или кортикостерона животным также снижает экспрессию Kiss1 в гипоталамусе [20][21].

В свете современных достижений молекулярной биологии и эндокринной гинекологии особую актуальность приобретают исследования, направленные на изучение локального распределения и концентрации нейропептидов непосредственно в тканях, что позволяет получить более полное представление о механизмах патогенеза репродуктивных нарушений. Так, в работе A.O. Abdelkareem и соавт. было продемонстрировано, что дефицит экспрессии кисспептина и KISS1R может быть ассоциирован с нарушением регуляции матриксных металлопротеиназ, сосудистого эндотелиального фактора роста и других сигнальных молекул, вовлеченных в процессы клеточной миграции, инвазии и ангиогенеза, что в совокупности приводит к нарушению ранней плацентации [22]. С другой стороны, нормогонадотропная форма аменореи — СПЯ — характеризуется персистирующей ановуляцией и повышенной продукцией андрогенов, что, в свою очередь, способствует формированию патологических изменений эндометрия, снижая его рецептивность и потенциально увеличивая риск репродуктивных потерь. Однако, как подчеркивают авторы, влияние овариальной гиперандрогении на экспрессию кисспептина в ткани яичников остается ­практически ­неизученным, что указывает на научную новизну и потенциальную значимость подобных исследований для выявления молекулярных мишеней при СПЯ и связанных с ним форм бесплодия. Дополнительные сведения той же исследовательской группы свидетельствуют о снижении экспрессии KISS1R в хорионических тканях эмбрионов как с эуплоидным, так и с анеуплоидным набором хромосом у пациенток с привычным невынашиванием беременности по сравнению с контрольной группой, что указывает на возможную роль сигнального пути кисспептина в механизмах ранней имплантации и формирования плаценты. Авторы подчеркивают, что для окончательного понимания причинно-следственных связей необходимо определить, является ли снижение активности кисспептиновой системы следствием или первопричиной нарушений плацентации [23]. Таким образом, изучение тканевой экспрессии нейропептидов открывает новые горизонты в понимании патофизиологии репродуктивных расстройств, включая СПЯ, ановуляцию и привычное невынашивание, а также закладывает основу для разработки диагностических и терапевтических стратегий, основанных на молекулярных мишенях.

У женщин с овариальной гиперандрогенией в крови выявлены повышенные уровни циркулирующего кисспептина, что указывает на его потенциальное участие в патогенезе данного состояния [24]. Отклонения в экспрессии нейропептида ассоциированы с дисрегуляцией гипоталамо-гипофизарно-гонадной оси, что, в свою очередь, усиливает пульсацию ЛГ, снижает уровень фолликулостимулирующего гормона и нарушает соотношение данных гонадотропинов [25]. Поскольку изучаемая молекула выступает центральным индуктором секреции ГнРГ и ЛГ, предполагается, что гиперэкспрессия кисспептина при СПЯ может быть причиной гормонального дисбаланса. В систематическом обзоре с метаанализом 2019 г. обнаружено статистически значимое повышение циркулирующего кисспептина у пациенток с СПЯ независимо от индекса массы тела [26], что подтверждается рядом других исследований [27].

Связь между кисспептиновой системой и ПНЯ представляет собой перспективное научное направление, особенно в свете недавних экспериментальных данных. В исследовании S.T. Ruohonen и соавт. у мышей с селективной элиминацией рецепторов к кисспептину в ооцитах наблюдались ановуляция, снижение уровня половых стероидов и признаки истощения овариального резерва, несмотря на сохранение гипоталамической экспрессии KISS1R и нормальные уровни гонадотропинов, что указывает на критическую роль периферических эффектов кисспептина в поддержании функции яичников [28].

Таким образом, работы, посвященные изучению кисспептиновой системы, открывают новые перспективы в понимании патогенеза аменорей различного генеза и могут способствовать разработке инновационных диагностических и терапевтических подходов для улучшения репродуктивного здоровья женщин.

## Нейротрофический фактор головного мозга

Нейротрофический фактор головного мозга (BDNF) — белок семейства нейротрофинов, экспрессируется в основном в нервной ткани. Основная функция BDNF заключается в индукции и контроле роста нейронов в процессе развития мозга. Доказано, что данный нейропептид участвует в возникновении возрастных патологий, когнитивных нарушений, расстройств пищевого поведения, депрессии и шизофрении [29]. Интересно, что BDNF также присутствует в фолликулярной жидкости, где он стимулирует созревание ооцитов [30].

BDNF является известным фактором роста, который продуцируют гранулезные клетки фолликулов. Его рецептор - TrkB, преимущественно экспрессируется в ооцитах, а взаимодействие BDNF с рецептором активирует сигнальный путь ERK1/2 [31][32]. Примечательно, что уровень экспрессии BDNF значительно ниже в фолликулах женщин в возрасте 61–64 лет по сравнению с более молодой группой участниц (29–35 лет), что указывает на возможную роль снижения его уровня в возрастном ухудшении качества ооцитов [33].

Вышеописанный нейротрофин в последние годы активно исследуют у женщин при гипоэстрогенных состояниях. Ряд независимых исследований продемонстрировал, что уровни BDNF в крови существенно снижаются у пациенток с ФГА и у женщин в постменопаузе, что позволяет рассматривать данный нейропептид как потенциальный маркер нарушения центральной и периферической нейроэндокринной регуляции [34–36].

В работе А. Podfigurna-Stopa и соавт. [34] было показано статистически значимое снижение концентрации BDNF у пациенток с ФГА по сравнению с женщинами репродуктивного возраста с нормальной менструальной функцией. Похожие результаты были получены и в более раннем исследовании S. Begliuomini и соавт. [35], в котором также было установлено, что уровень BDNF в сыворотке варьирует в зависимости от гормонального статуса и уровней половых стероидов. Кроме того, P. Drakopoulos и соавт. [36] обратили внимание на суточные колебания уровней этого белка у женщин с ФГА, что отражает тонкую циркадианную регуляцию нейротрофинов при функциональных расстройствах центрального происхождения.

В исследовании 2025 г. ученые под руководством J. Zhou указывают, что BDNF — ключевой активный компонент секретома мезенхимальных стромальных клеток, полученных из пуповины человека (hUC-MSC-sec), способный улучшать качество ооцитов и тем самым повышать потенциал их дальнейшего эмбрионального развития [37]. Данное утверждение было подтверждено экспериментом с добавлением блокирующего антитела против BDNF и специфического ингибитора рецептора TrkB в hUC-MSC-sec, что привело к значительному снижению частоты выброса первого полярного тельца (PB1) и увеличению числа аномальных митотических веретен. И напротив, введение BDNF значительно увеличивает частоту выброса PB1, снижает долю анеуплоидных ооцитов и способствует деградации материнской матричной рибонуклеиновой кислоты (мРНК) в стареющих ооцитах мышей in vitro. Дополнительно, положительные эффекты BDNF установлены в экспериментах in vivo, где наблюдалось улучшение формирования митотического веретена и развития эмбрионов, активируя сигнальный путь ERK1/2 [38].

Предполагается, что в период беременности плацента служит важным источником BDNF для плода, способствуя синтезу данного нейротрофического фактора, а также обеспечивая транспорт материнского нейропептида в кровоток [39]. Недавнее исследование ученых из Китая подтвердило ключевую роль BDNF в функционировании яичников, показав, что снижение его экспрессии в плаценте беременных мышей ассоциировано с обеднением овариального резерва у потомства уже в период внутриутробного развития, что позднее приводит к ПНЯ во взрослом возрасте. Это проявлялось в уменьшении числа гоноцитов и фолликулов, особенно примордиальных, у мышей в возрасте одной недели, одного, двух и четырех месяцев после рождения. У месячных мышей отмечалось уменьшение количества ооцитов, получаемых после стимуляции овуляции, что указывает на возможную нечувствительность животных к препаратам, используемых в протоколах вспомогательных репродуктивных технологий. Механизмы, с помощью которых BDNF способствует поддержанию жизнедеятельности яичников в долгосрочной перспективе, мало изучены. На основании известных функций BDNF в других типах клеток и тканях предположено, что нокаут гена Bdnf в плаценте потенциально нарушает пролиферацию первичных половых клеток посредством активации апоптоза в гоноцитах и повышения уязвимости последних к окислительному стрессу [40].

В работе польской группы исследователей под руководством A. Czyzyk и соавт. BDNF впервые был количественно оценен у пациенток с ПНЯ [41]. Согласно полученным данным, его концентрация составила 429,25±65,52 пг/мл, что было статистически значимо ниже по сравнению с контрольной группой здоровых женщин (479,75±34,75 пг/мл; p=0,0345). Несмотря на схожую тенденцию к снижению BDNF при ПНЯ и ФГА, стоит отметить различие в патогенезе этих состояний: если ФГА представляет собой обратимое гипогонадотропное расстройство, связанное с нарушением гипоталамической активности, то ПНЯ характеризуется органическим истощением фолликулярного аппарата и гипергонадотропным гипогонадизмом. Тем не менее, в обоих случаях выявлено снижение уровней BDNF, что может свидетельствовать о нарушении нейротрофической поддержки репродуктивной системы. Возможно, именно аменорея и является ключом к разрешению этого противоречивого факта, что наиболее подчеркивает важность и актуальность исследований, посвященных этой теме.

## Орексины

С момента открытия орексинов было установлено их участие в контроле репродуктивной нейроэндокринной оси, однако конкретные механизмы их действия в яичниках оставались до конца не изученными [42]. В последние годы растет научный интерес к роли орексинов в патогенезе гинекологических и эндокринных нарушений, что обусловлено их многофункциональностью и участием в регуляции как центральных, так и периферических процессов. Исследования в области эндокринной гинекологии рассматривают участие орексина-А в таких состояниях, как ФГА, СПЯ, эндометриоз, а также в нарушениях циркадианного ритма и сна, часто сопутствующих репродуктивным патологиям. Несмотря на функциональные связи орексинов с гипоталамо-гипофизарной регуляцией, большинство клинических исследований ограничиваются определением уровня орексина-А в периферической крови, что сопряжено с методологическими трудностями и ограниченной информативностью, учитывая их центральное происхождение и низкую концентрацию нейропептидов в сыворотке.

В работе E. Yilmaz и соавт. было показано, что у женщин с СПЯ уровень орексина-А в сыворотке крови статистически значимо ниже, чем у здоровых женщин контрольной группы. Более того, авторы обнаружили отрицательные корреляции между концентрацией орексина-А и рядом клинических и гормональных параметров, включая систолическое артериальное давление, гирсутное число по шкале Ферримана–Галлвея, уровни ЛГ и свободного тестостерона [43]. Предполагается, что снижение уровня данного орексина может быть связано с гиперандрогенией или инсулинорезистентностью, однако точные механизмы остаются неясными. Учитывая важную роль орексинов в регуляции энергетического обмена, аппетита и нейроэндокринной активности, необходимы дальнейшие исследования, направленные на выяснение того, является ли снижение концентрации орексина-А вторичным по отношению к метаболическим нарушениям при СПЯ, или же это звено первичной патогенетической цепи, влияющей на гонадотропную дисфункцию и клинические проявления синдрома [44].

Фундаментальные исследования, направленные на выявление молекулярных и клеточных механизмов регуляции репродуктивной системы, имеют важное значение для понимания патогенеза гинекологических заболеваний и разработки новых методов лечения женского бесплодия. В частности, работа M. Safdar и соавт. посвящена изучению орексина-A при апоптозе и пролиферации гранулезных клеток, а также механизмов, лежащих в основе его действия через сигнальные пути AKT/ERK1/2 и факторов, связанных с фолликулогенезом [45]. Показано, что орексин-А через взаимодействие с рецептором OX1R предотвращает запрограммированную гибель гранулезных клеток. Подавление экспрессии рецептора OX1R ведет к снижению экспрессии ядерного антигена пролиферирующих клеток (PCNA) и увеличению апоптоза гранулезоцитов посредством активации каспаз-3-зависимого пути и повышенной экспрессии проапоптотических факторов Bax и TNF-α с одновременным снижением антиапоптотического белка Bcl-2. Семейство пептидов Bcl-2 участвует в опосредованном митохондриями апоптотическом пути и рассматривается как ключевой фактор, контролирующий запрограммированную гибель половых клеток у женщин [46]. Кроме того, орексин-А влияет на клеточный цикл гранулезных клеток, предотвращая их задержку в S-фазе. Данный нейропептид опосредует экспрессию ооцитарных факторов BMP15 (костный морфогенетический белок 15) и GDF9 (фактор роста и дифференцировки 9), критически важных для нормального роста и созревания фолликулов [47]. Полученные данные указывают на значимую регуляторную роль орексина-А и его рецептора OX1R в поддержании физиологии яичников, подчеркивая значение этой сигнальной оси не только в фундаментальных аспектах репродуктивной биологии, но и в клинической практике. Это открывает перспективы использования орексина-А в качестве потенциальной терапевтической мишени для разработки новых стратегий коррекции женского бесплодия, вызванного нарушением процессов фолликулогенеза и овуляции.

## ЭКСПЕРИМЕНТАЛЬНОЕ МОДЕЛИРОВАНИЕ АМЕНОРЕИ: ВЫЯВЛЕНИЕ БАЗОВЫХ НЕЙРОЭНДОКРИННЫХ, МОЛЕКУЛЯРНЫХ И ФИЗИОЛОГИЧЕСКИХ МЕХАНИЗМОВ ПАТОГЕНЕЗА

Для изучения фундаментальных процессов возникновения и патогенеза аменореи с позиции нейроэндокринологии учеными используются лабораторные животные. Впервые подобная работа проведена F. Gaytan и соавт., в которой изучалась кисспептиновая сигнальная система в яичнике, дефект которой провоцирует развитие ПНЯ. У мышей с дефицитом KISS1R зарегистрировано преждевременное снижение частоты овуляции, за которым следовала прогрессирующая потеря антральных фолликулов, ооцитов и сокращение всех категорий преантральных фолликулов, что сопровождалось снижением фертильности. Далее у мышей в возрасте более 48 недель наблюдались атрофические яичники, отсутствие растущих фолликулов и желтых тел. Более того, авторы выявили, что нарушение фолликулогенеза и ановуляция, связанные с отсутствием KISS1R, не устраняются стимуляцией гонадотропинами. Полученные результаты свидетельствуют о наличии прямой овариальной роли кисспептина, реализуемой через аутокринно-паракринные механизмы, нарушение которых может способствовать развитию ПНЯ [48].

Примечательно исследование S.T. Ruohonen и соавт., в котором изучена передача сигнала кисспептина в ооците на лабораторных животных. В ходе эксперимента была создана линия мышей с абляцией KISS1R в ооцитах. У животных сохранялась инициация полового созревания при отсутствии клинических и морфологических признаков гипогонадизма. Однако уже в возрасте 2 месяцев у 40% мышей обнаружены гистологические признаки овариальной недостаточности и ановуляции. Пенетрантность фенотипа прогрессировала с возрастом, причем 80% и 100% самок показали полную овуляторную недостаточность со снижением уровня половых стероидов к 6 и 10 месяцам, соответственно, несмотря на неизменную экспрессию гипоталамического KISS1R и уровни гонадотропинов. Таким образом, нарушение регуляции сигналов кисспептина в ооците может быть основной и ранее незамеченной причиной некоторых форм ПНЯ у женщин [28].

В работе I. Velasco и соавт. проведен анализ функций кисспептина, экспрессируемого как совместно с нейрокинином В и динорфином, так и изолированно. Чтобы разделить физиологические эффекты нейропептида, полученного из разных субпопуляций кисспептиновых нейронов, была реализована условная абляция Kiss1 в KNDy-нейронах in vivo на лабораторных животных. У мышей наблюдалось снижение содержания кисспептина в аркуатном ядре гипоталамуса с более выраженным подавлением у самок, что было заметно в младенческо-пубертатном возрасте. Напротив, уровень нейрокинина В был полностью сохранен. Несмотря на снижение кисспептина в аркуатном ядре, старт полового созревания был нормальным у выведенных мышей обоих полов. Однако у молодых взрослых самок наблюдались нарушения пульсации ЛГ и уровня половых стероидов с подавлением базальной концентрации и предовуляторного пика ЛГ, снижение фертильности и развитие преждевременной овариальной недостаточности [49].

Перспективно исследование под руководством A.I. Ahmed и соавт., посвященное изучению роли некодирующих РНК (нкРНК) мезенхимальных стволовых клеток костного мозга (BM-MSC) и их взаимодействию с системой инсулиноподобный фактор роста 1 (ИФР-1) — кисспептин в экспериментальной модели ПНЯ, индуцированной циклофосфамидом (ЦФ) — гонадотоксичным алкилирующим агентом. Трансплантация BM-MSC крысам с моделью ПНЯ способствовала восстановлению активности гипоталамо-гипофизарно-яичниковой оси, что сопровождалось повышением экспрессии ИФР-1 и кисспептина в яичниках и гипоталамусе, снижением апоптоза гранулезных клеток, а также улучшением стероидогенеза, ангиогенеза, энергетического обмена и показателей окислительного стресса. Было показано, что BM-MSC экспрессируют повышенные уровни антиапоптотических длинных нкРНК и микроРНК, которые смягчали проявления овариальной недостаточности [50].

Создание экспериментальной модели ФГА имеет определенные сложности в связи особенностями ее этиологии. Известно, что этот тип аменореи возникает на фоне голодания, избыточных физических нагрузок, а также на фоне психоэмоциональных нарушений (например, при депрессии) [51]. Таким образом, возникают этические вопросы при воссоздании данных условий. Так как формирование условий психоэмоционального стресса на животных сложно осуществимо, то создать избыточную физическую нагрузку и голодание вполне реально. В своей работе L. Frintrop и соавт. разработали экспериментальную модель ФГА, при которой смертность лабораторных животных низкая. Крысы линии Wistar имели круглосуточный доступ к беговому колесу и получали 40% от своего базового потребления пищи до тех пор, пока не было достигнуто снижение веса на 20% или 25%. Далее этот вес тела поддерживался в течение двух недель, инициируя хроническое голодание. Полное отсутствие эстрального цикла было определено, как первичный исход. Резкое ограничение калорийности корма привело к нарушению цикла у 58% животных, а при хроническом голодании отмечалось его полное угасание [52].

Несмотря на наличие множества экспериментальных моделей СПЯ, большинство из них лишь частично воспроизводят характерные репродуктивные и метаболические фенотипы. У грызунов СПЯ моделируется с использованием различных подходов, включая пренатальное и постнатальное введение андрогенов, эстрогенов, ингибиторов ароматазы, высокожировой диеты и генетических манипуляций [53][54]. Наибольшее распространение получили модели, основанные на применении летрозола и высокожировой диеты, ввиду их способности воссоздавать морфологические, гормональные и метаболические особенности гиперандрогении овариального генеза [55]. Летрозол индуцирует ряд репродуктивных нарушений, типичных для СПЯ, включая увеличение массы тела, гипертекоз и ановуляцию [56], однако не вызывает выраженных метаболических сдвигов (инсулинорезистентность, ожирение, дислипидемия). Напротив, модели, основанные только на высокожировой диете, демонстрируют выраженные метаболические нарушения, аналогичные таковым у пациенток с метаболическим фенотипом СПЯ [57]. В исследовании S. Mirseyyed и соавт. разработана комбинированная модель синдрома на крысах линии Sprague Dawley с использованием летрозола и новой высокожировой диеты для оценки гормонального, метаболического, окислительного и ­воспалительного статуса. Наиболее выраженные эндокринные и метаболические нарушения отмечались в группе с комбинированным воздействием, включая повышение уровня андрогенов, нарушение углеводного и липидного обмена, транскрипционную гиперактивацию воспалительных сигнальных каскадов и биомаркеров окислительного стресса (активных форм кислорода и малонового альдегида) при снижении активности антиоксидантных ферментов [58]. Таким образом, совмещение терапии летрозолом с высококалорийным рационом индуцирует воспалительный ответ и окислительный стресс, формируя модель СПЯ, достоверно отражающую как гормональные, так и метаболические нарушения, характерные для клинической картины данного синдрома.

Создание экспериментальной модели ПНЯ сопряжено с рядом ограничений, обусловленных многообразием этиологических факторов, включая аутоиммунные, ятрогенные и генетические причины, при этом в клинической практике до 70% случаев остаются идиопатическими. Имитация аутоиммунного оофорита у лабораторных животных в рамках моделирования ПНЯ в настоящее время не представляется возможной. Таким образом, большинство моделей создаются путем резекции яичников или введения цитостатиков. D. Li и соавт. разработали модель ПНЯ у крыс путем двусторонней резекции яичников на 75% их объема. В отличие от полной овариэктомии, такая процедура не вызывала выраженного ухудшения общего состояния животных, но приводила к значимым нарушениям репродуктивной функции, что обеспечивало ее пригодность для последующих исследований. После операции отмечались сбои эстрального цикла, включая достоверное удлинение диэструса и снижение частоты овуляторных циклов (P<0,01), что подтверждает успешность моделирования ПНЯ [59].

В работе G. Lu для воспроизведения овариальной недостаточности у крыс использовали дозозависимое введение ЦФ. Самки были распределены на четыре группы: контроль (0,9% NaCl) и три экспериментальные (ПНЯ-1, ПНЯ-2, ПНЯ-3), получавшие ЦФ в дозах 25, 50 и 100 мг/кг в первый день, далее по 8 мг/кг в течение 14 дней. Эффективность моделирования оценивали по эстральному циклу, уровню стероидов, объему яичников и фолликулярному резерву. Наиболее выраженные нарушения наблюдались в группе ПНЯ-2, включая достоверное снижение массы яичников, истощение фолликулярного пула и морфологические признаки атрофии. Гистологический анализ подтвердил долговременные повреждения, включая потерю примордиальных фолликулов и атрезию. Умеренная доза ЦФ (50 мг/кг + 8 мг/кг × 14 дней) признана оптимальной для воспроизводимой индукции ПНЯ при сохранении высокой выживаемости животных, что делает данную модель релевантной по клинико-морфологическим параметрам [60].

## ЗАКЛЮЧЕНИЕ

Современные представления о нейроэндокринной регуляции репродуктивной функции свидетельствуют о сложной и многокомпонентной системе взаимодействия различных нейропептидов, обеспечивающих координацию процессов, критически важных для женского здоровья. Особую роль в поддержании нормального менструального цикла и фертильности играют кисспептин, BDNF, орексины и другие сигнальные молекулы, участвующие в регуляции продукции ГнРГ и энергетического обмена.

Кисспептин, являющийся ключевым активатором гонадотропной функции, обеспечивает запуск и поддержание секреции ГнРГ и, тем самым, регулирует гипоталамо-гипофизарно-гонадную ось. BDNF — молекула с выраженными нейропротективными и нейропластическими свойствами, принимает участие в развитии гипоталамических структур, вовлеченных в регуляцию женской половой системы. Орексины, наряду с участием в контроле бодрствования и энергетического баланса, играют важное значение в интеграции метаболических и гормональных сигналов. Нарушение скоординированного взаимодействия нейропептидов может приводить к устойчивым сбоям в работе репродуктивной оси, в частности к формированию различных форм аменореи.

Одной из серьезных проблем, ограничивающих возможности клинической интерпретации полученных данных, остается трудность точного определения уровней нейропептидов в системной циркуляции. Их низкие концентрации в периферической крови, высокая чувствительность к внешним воздействиям, быстрый метаболизм и вариабельность суточных колебаний существенно затрудняют использование этих молекул в качестве рутинных биомаркеров. В этой связи особую актуальность приобретают экспериментальные исследования на лабораторных животных, которые позволяют не только проследить каскадные механизмы регуляции, но и оценить влияние модификации нейропептидных сигналов на функциональное состояние репродуктивной системы. Такие модели создают основу для разработки новых диагностических подходов, направленных на раннюю идентификацию нарушений, а также открывают перспективы для таргетной терапии, способной модулировать активность нейропептидных путей.

Дальнейшее углубленное изучение нейропептидов как регуляторных и сигнальных молекул представляет собой важное направление в борьбе с ухудшением репродуктивного здоровья. Это особенно важно в контексте профилактики бесплодия, снижения сердечно-сосудистых рисков, связанных с гипоэстрогенными состояниями, и разработки стратегий активного женского долголетия. Интеграция фундаментальных знаний в клиническую практику позволит существенно расширить диагностические и терапевтические горизонты, способствуя сохранению и укреплению репродуктивного потенциала женщин в течение всей жизни.

## ДОПОЛНИТЕЛЬНАЯ ИНФОРМАЦИЯ

Источники финансирования. Работа выполнена в рамках государственного задания №123021300169-4 «Эпигенетические предикторы и метаболомная составляющая аменореи различного генеза у женщин репродуктивного возраста», 2023-2025 гг.

Конфликт интересов. Авторы декларируют отсутствие явных и потенциальных конфликтов интересов, связанных с содержанием настоящей статьи.

Участие авторов. Все авторы одобрили финальную версию статьи перед публикацией, выразили согласие нести ответственность за все аспекты работы, подразумевающую надлежащее изучение и решение вопросов, связанных с точностью или добросовестностью любой части работы.
